# Automatic Physiological Waveform Processing for fMRI Noise Correction and Analysis

**DOI:** 10.1371/journal.pone.0001751

**Published:** 2008-03-12

**Authors:** Daniel J. Kelley, Terrence R. Oakes, Larry L. Greischar, Moo K. Chung, John M. Ollinger, Andrew L. Alexander, Steven E. Shelton, Ned H. Kalin, Richard J. Davidson

**Affiliations:** 1 Waisman Laboratory for Brain Imaging and Behavior, Waisman Center, University of Wisconsin, Madison, Wisconsin, United States of America; 2 Neuroscience Training Program, Center for Neuroscience, University of Wisconsin School of Medicine and Public Health, Madison, Wisconsin, United States of America; 3 Medical Scientist Training Program, University of Wisconsin School of Medicine and Public Health, Madison, Wisconsin, United States of America; 4 Department of Psychiatry, University of Wisconsin, Madison, Wisconsin, United States of America,; University of Southern California, United States of America

## Abstract

Functional MRI resting state and connectivity studies of brain focus on neural fluctuations at low frequencies which share power with physiological fluctuations originating from lung and heart. Due to the lack of automated software to process physiological signals collected at high magnetic fields, a gap exists in the processing pathway between the acquisition of physiological data and its use in fMRI software for both physiological noise correction and functional analyses of brain activation and connectivity. To fill this gap, we developed an open source, physiological signal processing program, called PhysioNoise, in the python language. We tested its automated processing algorithms and dynamic signal visualization on resting monkey cardiac and respiratory waveforms. PhysioNoise consistently identifies physiological fluctuations for fMRI noise correction and also generates covariates for subsequent analyses of brain activation and connectivity.

## Introduction

**Figure 1:**
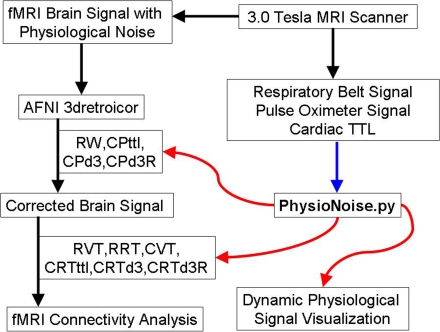
PhysioNoise Fills Physiological Signal Processing Gap. PhysioNoise processes physiological noise signals for existing software (AFNI) used in retrospective fMRI physiological noise correction and connectivity analyses. The inputs to PhysioNoise are represented by the blue arrow and the outputs from PhysioNoise are identified with the red arrows. (RW = Downsampled respiratory spline waveform; CPttl = Cardiac peak based on the TTL pulse from the scanner; CPd3 = Cardiac peak based on trough of the third derivative; CPd3R = Cardiac peak R-wave estimate based on small peak of third derivative preceding the CPd3; RVT = Respiratory volume over time based on peakdet peaks; RRT = Respiratory rate over time; CVT = Cardiac volume over time based on peakdet peaks; CRTttl = Cardiac rate over time based on the TTL pulse from the scanner; CRTd3 = Cardiac rate over time based on CPd3; CRTd3R = Cardiac rate over time based on CPd3R).

Physiological noise [1] describes a known low-frequency component of brain signals collected with functional MRI [2] (fMRI) that contaminates brain activation [3] and connectivity studies [4]. Retrospective removal of physiological noise from fMRI signal [5], [6] requires simultaneous collection of functional brain images along with cardiac and respiratory signals, typically using a pulse oximeter and a respiratory bellows belt, respectively. Further processing of the physiological signals is necessary to remove acquisition artifact and to prepare them for use in existing fMRI noise correction and analysis software like AFNI [7] [http://afni.nimh.nih.gov/afni/] . However, open-source software is not currently available to fill the gap between raw and processed physiological noise signals. We sought to fill this gap (Figure 1) by writing a fully automated, physiological noise processing program called PhysioNoise (download source code from Text S1) in the python language that enables interactive visualization of cardiac and respiratory time series, automatic detection of cardiac and respiratory peaks, and generation of processed cardiac and respiratory time series for both retrospective fMRI correction and covariates for analyses of brain activation and connectivity.

## Materials and Methods

### Algorithm

**Figure 2:**
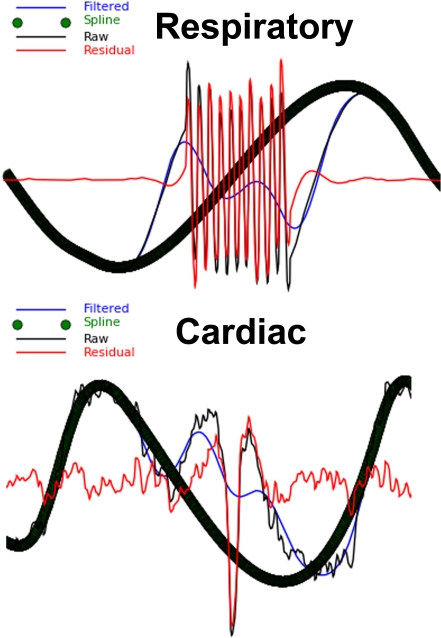
Plot of Physiological Signal Artifact. The raw respiratory and cardiac waveforms are plotted along with their filtered versions. Signal artifacts with large residuals (Raw Signal-Filtered Signal) are replaced with a spline interpolation. The series of green spline circles are the thick black waveform.

PhysioNoise was developed in python [8] using MacPython 2.5 [http://www.pythonmac.org/packages/] on the Mac OS X platform and requires scipy [9] [http://www.scipy.org/], numpy [10] [http://numpy.scipy.org/], and matplotlib [11] [http://matplotlib.sourceforge.net/] libraries. The program was also implemented and tested on a linux workstation running Scientific Linux [12]. PhysioNoise removes high frequency noise from both respiratory and cardiac waveforms with a user specified, zero phase, low pass Butterworth filter and calculates the residual (raw signal–Butterworth filtered signal). Acquisition artifacts are identified as time course regions with large residuals. A window around each artifact is removed and the entire filtered, cleaned dataset is fit with a spline such that clean peaks are modeled and the windowed gaps are replaced with values from the spline interpolation to produce a clean, spline waveform (Figure 2).

### Processing for Retrospective Physiological fMRI Noise Correction

#### Respiratory Waveform

Respiratory peaks are detected in the respiratory spline dataset (RW) using a slightly modified version of the open-source peakdet.m algorithm of Eli Billauer [13] which we translated to python. This algorithm alternately detects peaks and troughs in a periodic signal. We adapted this algorithm to detect respiratory (RPpd) and cardiac (CPpd) peaks by adding optional, user specified thresholds to limit peak detection to physiologically relevant timings and magnitudes. The downsampled respiratory spline waveform (RW) is output for use in retrospective fMRI noise correction software such as AFNI's 3dretroicor.

#### Cardiac Waveform

**Figure 3:**
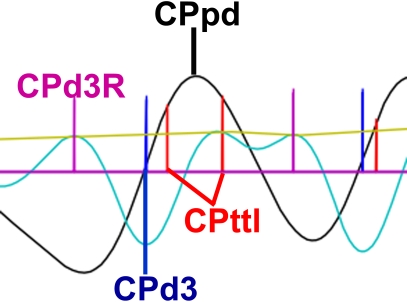
Cardiac Peaks. The CPpd, CPttl, CPd3, and CPd3R are displayed on the cardiac spline (black) and third derivative (cyan). The envelope of the third derivative peaks is shown in yellow. The plot also shows an example of the scanner TTL error of commission in which an extra CPttl was detected during one cardiac cycle. The differentiation method correctly produced one CPd3 peak (CPttl = Cardiac peak based on the TTL pulse from the scanner; CPd3 = Cardiac peak based on trough of the third derivative; CPd3R = Cardiac peak R-wave estimate based on small peak of third derivative preceding the CPd3).

The denoised cardiac spline was then subject to peak detection. For fMRI analysis there is a need to identify the ejection timing of the heart based on the pulse oximeter signal. The cardiac signal has several features that can be analyzed (Figure 3). A common method is to use the cardiac TTL pulse (CPttl) delivered by the scanner to estimate the R-wave, which is only detectable using EKG. Since the scanner records the peak times, PhysioNoise converts these times to a spike wave time series whose duration matches that of the fMRI brain scan. However, the CPttl peak detection contains artifactual errors of peak omission and commission and the CPttl onset time lags the R-wave [14]. Prior studies using Doppler measures of aortic flow confirmed that the timing of ventricle contraction and the onset of systole and diastole can be detected by taking the third derivative of the pulse oximeter waveform and are identifiable as two small peaks separated by a larger trough [15]. The trough is readily detectable even in noisy data, corresponds to the time when ventricular ejection occurs, and may be a closer approximation to R-wave onset that precedes the CPttl peaks. We identified the ventricular contraction time (CPd3) as the large trough of the cardiac waveform's third derivative. After implementation we determined that smaller troughs were detected which needed to be removed from CPd3. To do so, the envelope of the cardiac waveform's third derivative peaks was used to threshold the absolute value of the third derivative's troughs. Only troughs whose absolute magnitude was greater than that of the third derivative's peak envelope and at least half the mean envelope magnitude were reported as CPd3 peaks. The R-wave estimate (CPd3R) was then determined by selecting the nearest third derivative peak that preceded the large trough. The CPttl, CPd3, and CPd3R are saved to disk as options for retrospective fMRI correction.

### Processing for fMRI Functional Analysis of Activation and Connectivity

**Figure 4:**
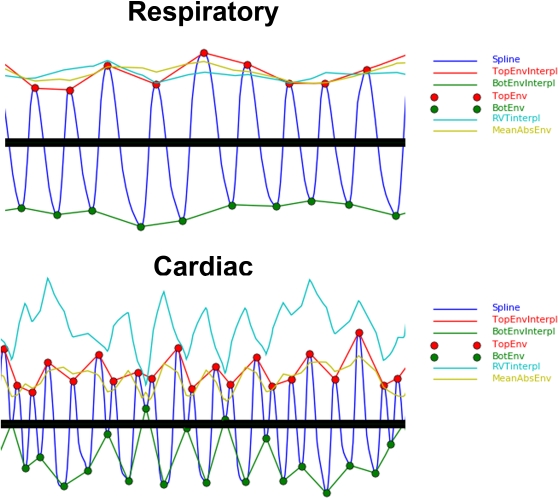
Respiratory and Cardiac Envelopes. The peaks identified by peakdet (RPpd,CPpd) are shown along with the respiratory and cardiac waveforms, top envelope, bottom envelope, and the absolute value of their means.

**Figure 5:**
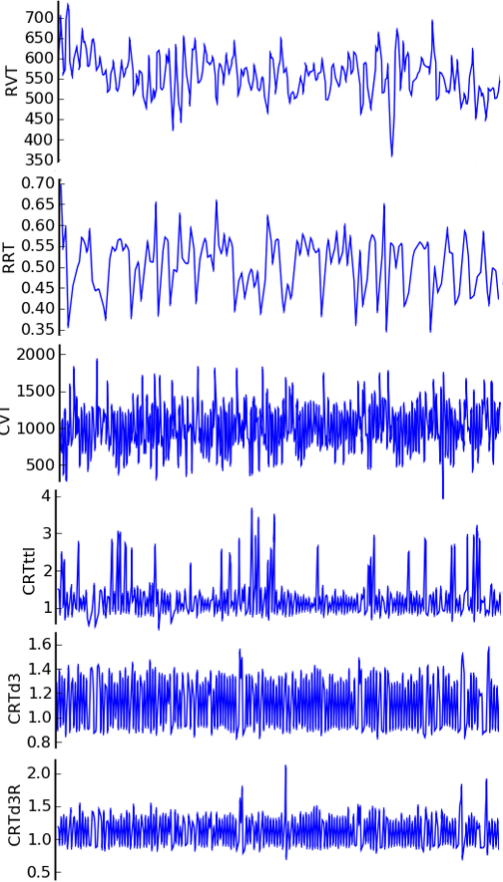
Cardiac and Respiratory Rates and Volumes over Time. Representative RVT, RRT, CVT, CRTttl, CRTd3, and CRTd3R waveforms are displayed in PhysioNoise. Note that the CRTd3 contains fewer outliers than the CRTttl and the CRTd3R. Dynamic visualization of these curves in PhysioNoise is possible using the zoom and scroll features (RVT = Respiratory volume over time based on peakdet peaks; RRT = Respiratory rate over time; CVT = Cardiac volume over time based on peakdet peaks; CRTttl = Cardiac rate over time based on the TTL pulse from the scanner; CRTd3 = Cardiac rate over time based on CPd3; CRTd3R = Cardiac rate over time based on CPd3R).

To prepare physiological noise covariates of no interest (nuisance variables) for connectivity analyses, further processing of the respiratory and cardiac peaks is required. First, the top and bottom respiratory envelopes and their difference, known as respiratory volume per time (RVT), are generated from their peaks (RPpd) as previously reported by Birn [16] using the peakdet algorithm. The cardiac volume per time curve (CVT) is also generated using peaks (CPpd) detected with peakdet algorithm (Figure 4). Second, cardiac rate time (CRT) courses based on the TTL (CRTttl), the third derivative (CRTd3), and the third derivative's R-wave estimate (CRTd3R) are generated as the inverse change in time between CP centered points on the initial peak [14]. The respiratory rate (RRT) over time was also calculated using the RPpd in the same manner as CRT (Figure 5). Third, the RVT and CRT waveforms are sampled on the TR and half TR and output for later use in fMRI activation and connectivity analyses.

## Results

With RARC approval, rhesus monkeys (n = 13) were sedated and restrained during simultaneous 3.0 Tesla GE Signa scanner acquisitions of functional MRI, pulse oximetry, and respiratory bellows signals. Respiration was monitored with a respiratory belt and cardiac contraction with a pulse oximeter on a 3.0 Tesla scanner (GE Medical Systems; Waukesha, WI). Both were collected at a 1000 Hz sampling rate. Artifacts in the physiological signal were automatically detected and corrected (Figure 2) using PhysioNoise prior to identifying RPpd, CPpd, CPttl, CPd3, and CPd3R (Figure 3). The downsampled signal and peaks were then available for use in retrospective fMRI physiological noise correction in AFNI. The respiratory and cardiac envelopes derived from the peakdet peaks of the spline waveforms (Figure 4) were successfully used to generate the RVT and CVT curves. The CRT curves were also successfully generated from the CPttl, CPd3, and CPd3R peaks. In short, PhysioNoise produced useful waveforms for fMRI analysis of activation or connectivity (Figure 1). Statistical tests were conducted in SPSS 14.0 (SPSS, Inc; Chicago) and R [17].

To evaluate the peakdet algorithm, we tested the null hypothesis that the period of both the respiratory and cardiac waveforms calculated from the peakdet peaks would be comparable to the period derived from the frequency of the maximum density in their respective power spectra. The temporal period (seconds) for the respiratory waveform determined by the peakdet algorithm [mean = 2.47; SD = 1.26] was comparable to the peak power [mean = 2.06; SD = 0.532; paired t(12) = 1.08; p = 0.301 ]. The temporal period of the cardiac waveform from the power spectra [mean = 0.868; SD = 0.133] was comparable to the period using CPpd peaks determined by peakdet [mean = 0.866; SD = 0.138; paired t(12) = −0.201; p = 0.844].

To evaluate the differentiation method for improved R-wave onset approximation, we compared the period from the CPttl peaks [mean = 0.805; SD = 0.225] to the period derived from the CPd3 [mean = 0.853; SD = 0.125] and CPd3R [mean = 0.853; SD = 0.133] peaks generated from the differentiation method and found them to be equivalent [F(2,24) = 0.989; p = 0.387] based on a one way ANOVA blocked across monkeys.

To determine whether CPd3 would provide a better estimate of R wave onset than CPttl, we evaluated the latency of CPd3 by testing the null hypothesis that the CPd3 time would be comparable to the CPttl onset. To do so, we measured the time change from CPd3R to the onset of both CPd3 [mean = 195.3 ms; SD = 51.1] and CPttl [mean = 232.2 ms; SD = 58.4] and took their difference. We determined that the CPd3 preceded CPttl by about 37 ms [mean = −36.8 ms; SD = 23.1] and this time lag was significantly different from zero [paired t(12) = −5.74; p = 0.00].

To quantify the spike reduction in the CRT waveforms, we tested the null hypothesis that the CRTttl, CRTd3, and CRTd3R would have comparable numbers of outliers (SD>4) and those outliers would have equal variance. The mean number of outliers for these methods were not equal [F(2,24) = 5.30; p = 0.012] when tested in a one way ANOVA blocked across monkeys. The CRTttl [mean = 6895; SD = 7228] had significantly more outliers than the CRTd3 [mean = 1662; SD = 3039; paired t(12) = 3.34; p  = 0.006] and marginally more significant outliers than CRTd3R [mean = 1963; SD = 3607; paired t(12) = 2.13; p = 0.055] methods. To demonstrate outlier dispersion, we tested the ratio of each method's outlier variances pooled across monkeys using an F-test. The CRTttl variance [mean = 0.312; SD = 0.833] was significantly more variable than the CRTd3 [mean = 0.036; SD = 0.068; F(1,24) = 6.37; p = 0.019] and the CRTd3R [mean = 0.026; SD = 0.046; F(1,24) = 15.6; p = 0.000].

## Discussion

PhysioNoise is a fully automated physiological noise processing program in the python language that processes physiological fluctuations and fills a gap between physiological noise signal acquisition and its use in fMRI neuroanalysis. Although PhysioNoise is fully automated, users have several options to control processing. Furthermore, PhysioNoise is open-source, modifiable by the users themselves, and may serve as a base program for further algorithm implementations. The utility of the program was validated using anesthetized monkey cardiac and respiratory signals. PhysioNoise detects and corrects acquisition artifact in the physiological signal and returns several processed waveforms suitable for fMRI correction and analysis.

PhysioNoise is available for download (Text S1) as a stand alone program. The open source algorithms we adapted as functions for use in PhysioNoise are contained within the PhysioNoise source code. Prerequisites to use PhysioNoise include a working installation of python [8] and access to scipy [9] [http://www.scipy.org/], numpy [10] [http://numpy.scipy.org/], and matplotlib [11] [http://matplotlib.sourceforge.net/] libraries. The PhysioNoise program was tested with physiological and fMRI signals collected from monkeys using a 3.0 Tesla GE Signa MRI scanner and a respiratory bellows belt. The algorithms we packaged into PhysioNoise are generalizable to other fMRI scanners and respiratory bellows equipment.

In summary, PhysioNoise outputs the RW, CPd3, CPd3R, and CPttl time courses as options for retrospective fMRI brain signal correction of physiological noise and the RVT, RRT, CVT, CRTd3, CRTd3R, and CRTttl curves as options for neuroanalysis. Prior studies have noted that the CPttl lags the R-wave by about 120 ms and produces CRT waves with intermittent spikes which are due to the detection of false peaks or omission of true peaks [14]. We detected the CPd3R about 230 ms prior to the CPttl R-wave estimate. This suggests that the CPd3R may precede the actual R-wave. Future studies should use PhysioNoise to directly compare the R-wave onset times from pulse oximetry using the differentiation method, the CPttl from the scanner, and the actual R-wave from EKG measurements during fMRI acquisition. The CPd3 may be a more appropriate choice for R-wave estimation for fMRI noise correction and analysis compared to the CPttl because the CPd3 preceded the CPttl by about 37 ms and the CRTd3 has fewer outliers with less outlier dispersion.

## Supporting Information

Text S1 PhysioNoise Source Code
(0.05 MB TXT
